# Minimally invasive sacroiliac joint fusion evidence gaps with biomechanics, devices, and industry influence: A narrative review

**DOI:** 10.1016/j.bas.2026.106163

**Published:** 2026-07-10

**Authors:** Jimmy Wen, Arsh Alam, Naomi Pai, Allyson Kouche, Shannon Dwyer, Sam Warner, Megan Diana Hsu, Jose Silva, Foad Elahi

**Affiliations:** a Highland Hospital, Department of Medicine, 1411 E 31st St, Oakland, CA, 94602, USA; b University of California, Davis, One Shields Avenue, Davis, CA, 95616, USA; c University of California, 110 Sproul Hall #5800, Berkeley, Berkeley, CA, 94720, USA; d Touro University California College of Osteopathic Medicine, 1310 Club Drive, Mare Island, Vallejo, CA, 94592, USA; e Sutter Roseville, Department of Emergency Medicine, 1 Medical Plaza Drive, Roseville, CA, 95661, USA; f California Center of Pain Medicine & Rehabilitation, 4944 Sunrise Blvd, Fair Oaks, CA, 95628, USA

**Keywords:** Sacroiliac joint, Stabilization, Lateral, Posterior, Posterolateral

## Abstract

**Introduction:**

Over the past decade, rapid growth in surgical approaches, implant designs, and industry involvement has expanded clinical adoption for minimally invasive sacroiliac joint fusion (SIJF), yet the supporting evidence remains unclear.

**Research question:**

This narrative review critically examines current literature on minimally invasive SIJF with emphasis on SIJ biomechanics, surgical approaches (lateral, posterior, and posterolateral), implant technologies, clinical outcomes, safety profiles, and market influences, while highlighting persistent evidence gaps.

**Material and methods:**

A narrative review was conducted using PubMed to evaluate studies reporting on clinical outcomes, device trends, biomechanics, and complications of minimally invasive SIJF.

**Results:**

The lateral transiliac approach is the most extensively studied and demonstrates consistent improvements in pain and function, though it carries higher reported complication rates due to proximity to neurovascular structures. Posterior and posterolateral approaches have gained popularity more recently, showing promising early clinical outcomes and lower complication rates, but are supported by fewer high-quality studies and shorter follow-up periods. Biomechanical investigations suggest that minimally invasive SIJF primarily achieves joint stabilization rather than complete arthrodesis, challenging traditional definitions of “fusion.”

**Discussion and conclusion:**

Across approaches, heterogeneity in study design, outcome measures, follow-up duration, and patient selection limits direct comparison and generalizability. High-quality randomized controlled trials with long-term follow-up, standardized outcome reporting, and direct comparisons among techniques are needed. Improved integration of biomechanics, patient-specific anatomy, and implant design will be essential to guide evidence-based decision-making and optimize patient outcomes in minimally invasive SIJF.

## Highlights

• SI joint fusion evidence is limited despite rapid procedural and device growth.
• True arthrodesis is unlikely and unproven due to anatomy and biomechanics.
• Device proliferation rapidly exceeds the available clinical evidence.
• Standardized criteria and independent trials are urgently needed.

## 1 Introduction

The sacroiliac (SI) joint is a large and complex load-bearing joint with minimal physiologic motion, extensive ligamentous support, and a fibrocartilaginous structure (Gartenberg et al., 2021). Diagnostic methods vary considerably, as the main reference standard is diagnostic injections, which inherently are subject to false positives and false negatives (McCormick et al.). Given this complexity, SI joint (SIJ) mediated low back pain (LBP) is one of the most challenging etiologies to understand and treat. Conservative treatment consists of non-steroidal anti-inflammatory drugs (NSAIDs), physical therapy, and intra-articular joint injections; however, one study showed that patients with conservative management alone experience minimal improvement (Raji et al., 2024). Minimally invasive SI joint fusion (SIJF) has become more widely adopted recently, with one study reporting a 2351% increase in utilization between 2010 and 2020 in the US (Federico et al., 2023). This is partly due to high market demand, with many implant systems obtaining Food and Drug Administration (FDA) clearance and a subsequent increase in the number of physicians incorporating these techniques into their practice. However, each device entered the market with limited or no independent clinical evidence base. Current reports show that the FDA has cleared over 30 SIJF implants through the 510k pathway, which requires only substantial evidence rather than demonstrated clinical efficacy (Kranenburg et al., 2022). This can lead to an environment where industrial and marketing forces take precedence over evidence-based practice.

However, despite this surge in use, the evidence base to support SIJF is limited. The inherent anatomical characteristics of the SIJ make a true arthrodesis very challenging. Current constructs used for SIJF include lateral trans-articular implants and posterior or posterolateral grafts/screws, which mainly function as stabilizers rather than true arthrodesis (Gheith et al., 2025). Long-term data on fusion rates, pain relief, and malunion/nonunion rates are not widely reported in the literature. A recent 2025 consensus guideline for carefully selected patients with SIJ pain stated that minimally invasive SIJF has weak or very weak evidence to provide benefit for at least one year (McCormick et al.).

Given these concerns, this narrative review aims to critically appraise and evaluate the literature on the rationale and evidence surrounding SIJF and the disconnect between the theoretical fusion mechanisms and anatomical plausibility. We dive into the SIJ anatomy, mechanisms of the three main types of SIJF, clinical outcomes, and industry developments.

## 2 Literature search methods

A focused literature search was performed in PubMed from inception through January 2026 for studies evaluating the anatomy, biomechanics, clinical outcomes, and safety profiles of lateral, posterior, and posterolateral approaches of SIJF. The search strategy encompasses the following keywords alone or in combination with the MeSH terms (OR, AND): sacroiliac joint fusion; SI joint fusion, sacroiliac joint arthrodesis, sacroiliac joint fixation, minimally invasive sacroiliac joint fusion, lateral SI joint fusion, posterior SI joint fusion, posterolateral SI joint fusion, fusion rates, clinical outcomes, and safety.

Studies were included if they met the criteria: (1) published in English, (2) covered the anatomy, biomechanics, technique, clinical outcomes, or safety of lateral, posterior, or posterolateral SIJF, (3) studies were in cadavers, human patients, or biomechanical testing. Exclusion criteria consisted of non-SIJ studies, review articles, animal studies, editorials, and commentaries.

### 2.1 Anatomy of the SI joint

The SIJs are the articulations between the sacrum and the ilia on both sides of the body. They form the transition between the spine and pelvis, playing a critical role in providing stability and load transfer from the trunk to the lower limbs. Each SIJ is a diarthrodial synovial joint consisting of a joint space filled with synovial fluid and surrounded by a fibrous capsule. A distinguishing feature of diarthrodial synovial joints is that the articulation occurs between two different types of cartilage. The sacral articular surface consists of hyaline cartilage, while the iliac capsular surface is composed of fibrocartilage (Wong et al., 2025). The hyaline cartilage on the sacral surface reaches approximately 4 mm in thickness, whereas the opposing iliac surface has a thinner layer of fibrocartilage measuring about 1 to 2 mm (Cho and Kwak, 2021). Movement within the SIJ is limited due to several factors, including the wedge-shaped configuration of the sacrum and the extensive ligamentous support surrounding the joint (Cho and Kwak, 2021).

The SIJ typically involves the S1, S2, and S3 segments of the sacrum, although S3 is often not fully involved in females. These segments form three regions of the joint, commonly referred to as the cranial, middle, and caudal portions. Despite significant individual variation, the sacral auricular surface is generally concave, while the iliac surface is convex. This configuration promotes bony congruency and joint stability. Additional stability is provided by interdigitating symmetrical grooves and ridges on the articular surfaces. These features increase the coefficient of friction and help resist translational movement. The overall shape of the sacrum also contributes to stability, as it is wider superiorly than inferiorly and wider anteriorly than posteriorly. This allows the sacrum to fit securely within the pelvic ring to help resist shearing forces generated by vertical compression (Vleeming et al., 2012).

Several primary ligaments, along with smaller accessory ligaments, contribute to the stabilization of the SIJ. The major stabilizing ligaments include the interosseous SI ligament, posterior SI ligament, anterior SI ligament, sacrotuberous ligament, and sacrospinous ligament (Vleeming et al., 2012). Among these, the interosseous SI ligament is the strongest and provides multidirectional stability. Additional functions of the ligaments include stabilizing the lumbar vertebrae on the sacral base, limiting sagittal plane motion at the joint, and assisting with the transfer of mechanical forces between the trunk and lower extremities (Vleeming et al., 2012).

The nerve supply of the SIJ is complex. Current evidence suggests that innervation is provided by small nerve branches arising from the dorsal rami of spinal nerves S1 through S4 (Vleeming et al., 2012). Some studies have also reported contributions from additional sources, such as the L5 nerve root, although S1 through S4 remain the most consistently identified contributors. Various nerve fiber types are present within the joint and exhibit physiological properties that are likely associated with SIJ pain (Vleeming et al., 2012).

An important consideration when examining SIJ anatomy is the significant natural variability in bony morphology. Variations exist not only between individuals but also between the left and right joints within the same person. Differences in joint shape and surface contour are common and are particularly notable in females (Jurik and Herregods, 2024). Numerous normal morphological variants have been described, including differences in articular surfaces, the iliosacral complex, secondary ossification centers, bipartite and crescent bony plates, semicircular defects, and the presence of accessory joints (Bovinet et al., 2025a). Certain populations, such as elderly adults, individuals with obesity, females, and those with mechanical SIJ disorders, are more likely to experience these variations. A population-based imaging study indicated that approximately 25.7-35.7% of individuals demonstrate some form of SIJ variation, with some studies reporting a prevalence as high as 52.2% and 82.8% (Bovinet et al., 2025a). Awareness of these common normal variants is important when examining SIJ anatomy, as they can influence biomechanics and should not be mistaken for pathology (Jurik and Herregods, 2024).

### 2.2 Diagnostic tests for SI joint pain

The accurate diagnosis for SIJ-mediated pain remains a challenge as clinical symptoms often overlap with those from the thoracolumbar spine, hip, and pelvic organs (Raj et al., 2025). Careful focus on the history, pain localization, gait pattern, physical exam, and imaging is required to determine if the SIJ is the source of pain. Positive findings from at least 3 physical exam maneuvers that stress the SIJ are required for diagnosis (e.g., FABER/Patrick's test, Gaenslen test, Yeoman test, distraction test, compression test, sacral thrust, and thigh thrust) (Buchanan et al., 2021). Based on these maneuvers, three or more of these maneuvers have a 91% sensitivity and 78% specificity for SIJ pain (Buchanan et al., 2021). Vanaclocha et al. evaluated the diagnostic accuracy of SIJ pain among 364 patients and found that the lowest accuracy was with medical history only (85-86%), higher with radiography added (87%), and highest when physical exams were included (96%) (Vanaclocha et al., 2026). Imaging alone can identify structural abnormalities or alternative pain generators, but it is often not enough to definitively diagnose SIJ-mediated pain. Image-guided intra-articular diagnostic anesthetic injections are typically considered the reference standard, and temporary pain relief supports the diagnosis of the SIJ as the primary pain generator (Raj et al., 2025). The current consensus practice guidelines from McCormick et al. report that the cutoff for positive diagnostic/prognostic blocks is commonly set at 50%, with some. Similarly, some societies believe that selecting patients for minimally invasive SIJF should be based on greater than 50% pain relief with documented functional improvement after a single block (McCormick et al.).

## 3 Mechanisms of different SI joint fusions

SIJF is indicated for patients with chronic SIJ pain and instability. This procedure includes three main approaches: lateral, posterolateral, and posterior. The choice of approach is dependent on physician preference and patient-specific anatomy. The lateral approach accesses the SIJ via a small incision on the lateral side and uses a bone graft/screw/rod for fixation. The posterior approach is newer and involves a small posterior incision, decorticating the joint, placement of an intra-articular bone graft, and fixation with screw/rods inside the joint line (Bovinet et al., 2025a). The posterolateral approach involves the upper outer surface of the iliac crest, enabling a more direct course to the SIJ through the ilium (Cahueque et al., 2023). No matter the approach, preoperative planning with computed tomographic (CT) imaging of the SIJ is essential to determine the appropriate implant size and trajectory depending on the patient's anatomy and bone quality (Cahueque et al., 2023).

### 3.1 Lateral

**Fig. 1:**
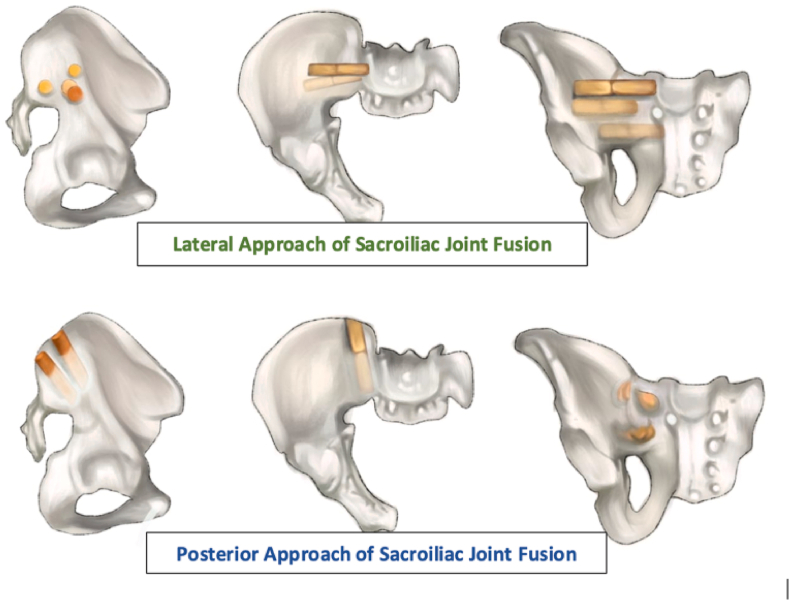
Schematic depiction of the lateral and posterior approaches for minimally invasive sacroiliac joint fusion.

Reproduced from Medani et al. (Abbasi et al., 2025) under the license CC BY-NC-ND.

The lateral approach for SIJF is the most established method, typically performed with a triangular titanium implant (TTI) featuring a plasma-sprayed porous surface (with a porosity similar to that of cancellous bone) to achieve transarticular stabilization and long-term biological fixation of the SIJ (Fig. 1) (Abbasi et al., 2025).

However, this approach inherently crosses major neurovascular structures (Gill et al., 2025). This procedure is performed in the prone position under general anesthesia, utilizing fluoroscopy or CT imaging for navigation (Polly and Holton, 2020). A probe is used to approximate the desired location of entry and trajectory. These positions are then marked on the skin, and a 3 to 5 cm lateral incision is made. Blunt dissection of the gluteal fascia is performed on the outer ilium. A guide pin is then passed across the SIJ and into the center of the sacrum on the lateral side of the neuroforamina. This area is then drilled and broached, followed by three TTIs using a pin-guide system (two in S1 and one in S2). The upper implant is positioned in the ala, the middle implant is positioned above or next to S1, and the bottom implant is placed between the S1 and S2 foramina (Medani et al., 2025). Multiplanar imaging after placement is performed before wound irrigation and closure (Polly and Holton, 2020).

In 2017, a 3D-printed TTI was cleared by the FDA. 3D printing enables the creation of a custom porous surface with internal fenestrations (without the need for plasma spray coating), aiming to accelerate and improve osseointegration into the SIJ (Patel et al., 2025). Patel et al. found similar improvements in pain, disability, and quality of life compared to standard porous-coated TTIs (Patel et al., 2025). At the five-year follow-up, radiologically, all implants showed osseous integration, 87% had bridging bone, and no implants exhibited signs of loosening. Interestingly, in some cases, decortication of the joint was performed, and demineralized bone matrix (DBM) was inserted before insertion of the TTI. Still, radiographic results were similar to those when DBM was not used.

More recently, 3D-printed porous threaded titanium implants (PTI) with similar characteristics as TTIs but in a screw design have been used (Jung and Kuhns, 2025). The PTI likewise has a lattice surface with a porosity similar to cancellous bone. In addition, this design contains helical flutes that self-harvest bone during insertion, which feeds the autograft into the cannulated interior of the implant through its fenestrations (osseointegration) (Jung and Kuhns, 2025).

A novel approach to lateral SIJF by Abbasi et al. was placing hollowly fenestrated screws in a tri-pronged fashion across the joint (Abbasi et al., 2025). This system of screw placement and fixation is performed once rather than requiring repeat surgical entry, which can reduce the risk of neurovascular injury. Other types of implants that have been used are the triangular dowel implant (TDI) and the cylindrical threaded implants (CDI).

### 3.2 Posterior

The posterior approach was developed and proposed as less invasive and safer than the lateral approach, as it avoids the neurovascular bundle (Fig. 1). This approach varies depending on the type of implant and the technique used. For example, one utilizes two biologic implants in orthogonal positioning, one utilizes 1-3 cylindrical implants, and one uses a cortical bone allograft implant (Bovinet et al., 2025a). There are two mechanisms for the posterior approach: surgical screw fixation and percutaneous graft placement.

The surgical screw fixation posterior approach is performed by positioning laterally from the posterior superior iliac spine (PSIS) towards the sacral promontory. Fluoroscopy is positioned to obtain imaging of the sacral outlet to locate the area between the S1 and S2 foramina where the implants will be placed (Medani et al., 2025). A segment of the SIJ ligament is also usually excised. The graft is targeted between S1 and S2 while avoiding any lower placements based on biomechanical study findings (Moghim et al., 2025). A pedicle access kit (PAK) is then inserted through the ilium into the SIJ and then into the sacrum. The PAK is then exchanged with a guidewire, and then drilling is performed. Following this, a threaded implant is inserted through the channel and crosses the SIJ until the head is aligned with the ilium's surface. These steps can be repeated as needed up to two times, for a total of three implants (Medani et al., 2025).

In the percutaneous graft placement, fluoroscopy is tilted at 15-20° (medial to lateral oblique) until the posterior and anterior SIJ lines overlap (Kaye et al., 2024). Then, a Steinman pin(s) is inserted into the SIJ to place one or two allografts. Following this, either the joint is decorticated or a surgical drill is used through the retraction tube and DBM, and the cortical allograft(s) are placed into the SIJ (Kaye et al., 2024).

A newer method using an inferior intra-articular approach has also been described. This trajectory aligns the allograft implant below and towards the front of the PSIS, within the articular part of the joint, perpendicular to the S1 endplate (Kaye et al., 2024).

Preliminary evidence suggests that the use of an allograft leads to improved pain scores compared to SIJF without an allograft. It has been proposed that the allograft may lead to greater stability of the fused SIJ []. Cadaveric biomechanical studies have shown a reduction in range of motion in all planes while aligning the center of the axis of rotation to the implant to facilitate graft fusion (Bovinet et al., 2025a).

### 3.3 Posterolateral

**Fig. 2:**
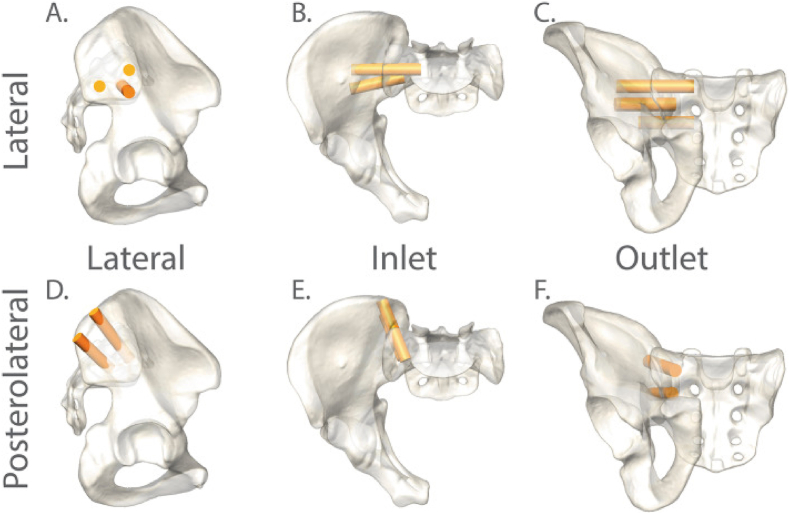
Schematic depiction of the lateral and posterolateral approaches for minimally invasive sacroiliac joint fusion.

Reproduced from Payne et al. (Jung and Kuhns, 2025), under the license CC BY-4.0.

The posterolateral or posterior-oblique approach is another promising new technique for SIJF. An advantage compared to the lateral or posterior approach is that it avoids dissecting through gluteal tissue, SIJ ligaments, risk of injury to the cluneal nerves and S1 and S2 within the foramina, and the branches of the superior gluteal artery (Fig. 2) (Kaye et al., 2024; Payne et al., 2020).

This approach is performed prone under general anesthesia (Cahueque et al., 2023). Fluoroscopy in lateral, outlet-oblique, and inlet views is used to mark the superior edge of the sacral ala transversely and the lateral edge of the ilium vertically. Needle insertion is approximately one finger width below the superior edge of the ala and one finger width lateral to the ilium. The needle is then positioned at an angle of approximately 5-20° relative to the horizontal plane, aiming for the upper outer surface of the iliac crest and along the superior surface of the ala. The needle is then guided through the ilium and SIJ and towards the anterior superior corner of the ala. The inner style of the needle is then removed, and a guidewire is advanced until the tip reaches past the tip of the bone needle. A 1.5 cm longitudinal incision is made below the guidewire, and a tissue dilator is introduced. After the tissue dilator is removed, a self-tapping screw embedded with the bone graft is directed towards the anterior superior surface of the ala. A second implant is placed 1.5 cm below the first implant using the same incision in a parallel fashion (Cahueque et al., 2023). Preliminary evidence suggests that the posterolateral approach allows for surface-bridging with screw fixation, which reduces joint movement. In addition, due to less soft tissue manipulation and muscle dissection, this may facilitate shorter recovery times (Cahueque et al., 2023).

The posterolateral transiliac (PLTI) approach involves initiation at a more posterior position along an angled trajectory across the SIJ (Kaye et al., 2024). The bone surface is decorticated, and a drill/broach is then used to carve out the channel in the subchondral bone. This theoretically facilitates a secure intra-articular placement of the allograft along the joint line to increase stability. The positioning of the implant below the PSIS and along the sacral axis of rotation may decrease biomechanical forces with sacral flexion-extension and rotation (Kaye et al., 2024).

A summary of the characteristics of the three approaches can be found in Table 1.

**Table 1:** Comparison between SIJF approaches.

Approach	Mechanism	Advantages	Limitations
Lateral	Transarticular Fixation	Most studied approach, biomechanically stable fixation	Crosses neurovascular structures, technically difficult in certain anatomical situations, and true arthrodesis evidence is uncertain
Posterior	Intra-articular graft or screw fixation	Minimally invasive, avoids the neurovascular bundle	Graft migration risks, potentially lower biomechanical rigidity, limited high-quality studies, and unclear long-term outcomes
Posterolateral	Screw fixation	Avoids extensive dissection through soft tissues, familiar fixation concept	Least amount of literature available, risk of implant loosening/migration, unclear long-term outcomes

## 4 Clinical evidence and safety of SI joint fusions

### 4.1 Clinical outcomes

The lateral transiliac approach has been the most studied technique for SIJF, and several studies have supported its effectiveness in reducing pain. In contrast, the posterior approach is still relatively new, with few studies available to examine its safety and efficacy. Though supported by a substantially smaller evidence base, this technique has demonstrated clinical outcomes comparable to the lateral approach. Similar to the posterior approach, the posterolateral technique is a newer and less extensively studied approach; nevertheless, early clinical outcomes are favorable. A 2025 meta-analysis by Xu et al. reported pooled mean differences between preoperative and postoperative visual analog scale (VAS) scores for LBP across 48 studies. At 6 months postoperatively, the pooled mean difference was 4.3 (95% CI 3.6, 5.0; 9 studies) for the lateral approach, 4.8 (95% CI 3.6, 6.0; 3 studies) for the posterior approach, and 3.0 (95% CI 1.6, 4.4; 3 studies) for the posterolateral approach (Xu et al., 2025). At 12 months, there was a pooled mean difference of 5.0 (95% CI 4.5, 5.4; 15 studies) for the lateral approach, 4.9 (95% CI 3.6, 6.2; 2 studies) for the posterior approach, and 3.8 (95% CI 1.9, 5.7; 2 studies) for the posterolateral approach (Xu et al., 2025).

These short- and mid-term outcomes are complemented by longer-term data from a prospective trial by Patel et al. (2024), which followed 51 subjects, 46 with unilateral and 5 with staged bilateral SIJF, for up to 60 months. The subjects experienced significant improvements in SIJ pain, with mean pain scores decreasing from 79 ± 11 at baseline to 21 ± 26 at final follow-up (Patel et al., 2025). Furthermore, the researchers reported an improvement in Oswestry Disability Index (ODI) scores by an average of 25 points (53.0 to 27.9), and an improvement in quality of life, with EQ-5D scores increasing from 0.47 to 0.76 (Patel et al., 2025). Notably, over 80% of patients experienced a reduction of at least 20 points in pain scores across all time points (Patel et al., 2025). Notably, in a 6-year comparative case series with conservative management, SIJ denervation, and SIJF, patients undergoing SIJF had the largest improvements in SIJ pain (mean 6 points), disability (mean 25 points), opioid use, and final work status (Vanaclocha et al., 2018a). Mechanistically, areas of increased bone density have been observed in parts of the ilium and sacrum adjacent to implant sites following SIJF (Patel et al., 2025). This suggests an enhanced load transfer through the fusion site. These findings are consistent with Wolff's law and empirical work by Poillot; thus, the implanted bone may be bearing a mechanical load that was previously dissipated across the SIJ and the surrounding soft tissues. This load sharing is perhaps an underlying factor in the pain relief seen in patients (Patel et al., 2025; Wolff, 2010; Poilliot et al., 2021).

For the posterior approach, Bovinet et al. conducted a multicenter study in 2025 involving 258 patients undergoing posterior SIJF. Patients showed a fairly rapid increase in numerical rating score (NRS) scores from a baseline of 7.61 ± 1.64 to 3.54 ± 2.45 at 2-week follow-up. At final follow-up of 20.9 months, the cohort had a mean NRS score of 1.60 ± 1.86. These improvements were statistically and clinically significant (Bovinet et al., 2025b). In a separate 2025 retrospective cohort study by the same author, NRS scores and opioid usage were recorded for 208 patients. Bovinet et al. reported a similar improvement of the average pain score from 7.23 ± 1.82 (preoperative) to 0.95 ± 1.55 (postoperative). At the final follow-up of six months, the mean pain score was 1.16 ± 1.35 (Bovinet et al., 2025a). Additionally, subgroup analyses showed a decrease in the mean NRS score from 7.22 ± 1.82 to 2.72 ± 2.47 at 6 weeks (n = 201) and 7.31 ± 1.69 to 1.00 ± 1.19 at six months (n = 153). Opioid usage was also reported as decreasing from a mean morphine milligram equivalents (MME) consumption of 20.74 ± 26.33 mg at baseline to 10.00 ± 18.59 mg at final available follow-up (Bovinet et al., 2025a). Overall, there was an 84% average reduction in pain levels and a 51.8% decrease in opioid consumption at final follow-up, and these changes were statistically significant (p < 0.001) (Bovinet et al., 2025a). Notably, this study employed preoperative CT mapping, which may have contributed to the favorable clinical outcomes observed in both pain relief and reduced opioid consumption. Additional observational studies, including those by Sayed and Lynch, have documented functional improvements and pain relief in patients receiving intra-articular allograft placement, particularly in those with prior failed lateral fusions. A 2022 case series reported outcomes in seven patients with persistent symptoms at 6 months or more after lateral SIJF, followed by articular allograft implantation via a posterior approach. Notably, all patients reported at least a 50% reduction in pain scores and a 75% improvement in NRS scores at a mean follow-up of 9.8 months (Gill et al., 2025; Sayed et al., 2022). In another 2022 study, 57 patients received a fluoroscopically guided posterior intra-articular allograft placement. At 6 months follow-up, the researchers found statistically significant improvements in pain-tolerant walking and standing with no reported complications (Xu et al., 2025; Lynch et al., 2022). Interestingly, in a multi-center cohort study involving 20 hospitals, 73% of patients reported feeling better or much better, but only 49% reported reducing their pain medication intake at 24 months post-procedure (Fuchs and Ruhl, 2018).

In a 2022 retrospective analysis of 43 patients undergoing posterolateral SIJF, Sarkar et al. reported a statistically significant 81% mean improvement in VAS scores at a 12-month follow-up, with 3 patients reporting no change in pain (Sarkar et al., 2022). In a 2023 case series, Chin et al. reported pain outcomes using the VAS scale in 3 patients who underwent salvage fusion following lateral SIJF. The researchers found an 89% decrease in pain from the mean preoperative VAS score following placement of two variable-threaded screws via posterolateral oblique trajectory (Chin et al., 2023).

A summary table of the main findings across the three approaches can be found in Table 2.

**Table 2:** Summary of main clinical outcomes for SIJF approaches.

Study	Approach	Design	Number of Patients (n)	Follow-up (Months)	Main Findings
Xu et al., 2025	Lateral, Posterior, Posterolateral	Meta-Analysis	Lateral (1906) Posterior (667) Posterolateral (333)	12	Pooled mean difference: Lateral (5.0) Posterior (4.9) Posterolateral (3.8)
Patel et al., 2024	Lateral	Prospective	51	60	Mean pain scores decreased from 79 ± 11 at baseline to 21 ± 26 at final follow-up. Increase in ODI by an average of 25 points (53.0 to 27.9).
Bovinet et al., 2025	Posterior	Retrospective	258	20.9	Decrease in NRS scores from a baseline of 7.61 ± 1.64 to 1.60 ± 1.86.
Bovinet et al., 2025	Posterior	Retrospective	208	6	Average pain score decreased from a base line of 7.23 ± 1.82 to 1.16 ± 1.35. Opioid usage decreased from a mean MME consumption of 20.74 ± 26.33 mg to 10.00 ± 18.59 mg.
Sayed et al., 2022	Posterior	Case Series	7	9.8	At least 50% pain score reduction and 75% improvements in NRS scores
Fuchs and Ruhl, 2018	Posterior	Prospective	171	24	73% reported feeling better or much better. 49% reduced their pain medication intake.
Sarker et al., 2022	Posterolateral	Retrospective	43	12	81% mean improvement in VAS scores.
Chin et al., 2023	Posterolateral	Case Series	3	19.3	89% decrease in pain from the mean preoperative VAS score.

### 4.2 Safety

In terms of safety, Xu et al. reported a total complication rate of 9.2% (95% CI 4.4%, 15.2%; 16 studies), 1% (95% CI 0.1%, 2.6%; 6 studies) for the posterior approach, and 3.7% (95% CI 0.0%, 21.0%; 3 studies) for the posterolateral approach (Jung and Kuhns, 2025). Furthermore, pooled revision rates were 2.4% (95% CI 1.3%, 3.9%; 28 studies) for the lateral approach, 0.6% (95% CI 0.0%, 1.8%; 6 studies) for the posterior approach, and 0.9% (95% CI 0.0%, 2.9%; 3 studies) for the posterolateral approach (Xu et al., 2025). Seven studies on the lateral approach reported a pooled fusion rate of 88.1% (95% CI 76.7%, 96.4%) (Xu et al., 2025). In contrast, 4 studies reported a fairly low pooled fusion rate of 66.9% (95% CI 29.9%, 95.5%) for the posterior approach. However, after excluding a 2018 study by Fuchs et al. (fusion rate of 31%) to reduce heterogeneity, the pooled fusion rate was recalculated to be 83.1% (95% CI 69.5%, 93.8%) (Xu et al., 2025; Dengler et al., 2017). Finally, 4 studies on the posterolateral approach reported a fairly high pooled fusion rate of 95.2% (95% CI 84.7%, 100.0%) (Xu et al., 2025). Though promising, it is important to consider the limited evidence base for the posterolateral approach. While the previously presented results suggest strong early efficacy, the evidence base remains narrow, and many studies lack rigorous design, large sample sizes, or extended follow-up.

In addition to the pooled complication and revision rates reported by Xu et al., further safety insights are available from individual trials. In a prospective, multicenter randomized controlled trials (RCT), Dengler et al. reported 1- and 2-year follow-up data for minimally invasive SIJF performed via a lateral approach. The researchers found that 4 of 39 severe adverse events were procedure-related (post-procedural pain, hematoma, and nerve impingement). Furthermore, cortical breach occurred in 8.6% of cases, although most were clinically insignificant (Dengler et al., 2017). In a retrospective cohort using TTI, Smith et al. reported no intraoperative issues in 114 patients. However, there were still postoperative events such as falls, facet joint pain, and cellulitis present (Smith et al., 2013). Among 27 cases, Ledonio et al. reported 2 revisions due to implant loosening and 1 pulmonary embolus, and Vanaclocha et al. reported transient sciatic symptoms with no need for reoperation (Ledonio et al., 2014; Vanaclocha et al., 2018b). Though rare, serious vascular complications have been reported in case studies. For example, a case report by Maxwell et al. described a superior gluteal artery injury following percutaneous lateral SIJ fusion, which required open revision and branch ligation (Maxwell et al., 2021). Additional retrospective case series focusing on lateral screw-based techniques provide further insight into complications associated with the lateral approach. Furthermore, in 18 patients, Kube & Muir reported 1 guide wire fracture, 1 case of prolonged postoperative pain, and 2 cases of prolonged surgery time, with minimal postoperative complications overall (Kube and Muir, 2016). Similarly, in a case series with 24 patients, Rajpal & Burneikiene reported relatively minor adverse events, such as 2 spontaneously resolving subcutaneous hematomas, 2 superficial wound infections, and 1 osteophyte adjacent to the implant (Rajpal and Burneikiene, 2019).

Additional prospective and observational studies support the safety profile of posterior approaches. Endres and Ludwig did not report any complications in their prospective case series (n = 19) using a posterior screw-based technique (Endres and Ludwig, 2013). Similarly, a study of 111 patients treated with a minimally invasive posterior implant did not report any complications (Deer et al., 2021). Furthermore, in a series of 50 patients with a follow-up of 12 months, only 1 case of device migration caused the need for revision (Sayed et al., 2021). Finally, a large multicenter prospective study of 122 participants reported 4 serious adverse events, with only 1 being procedure-related, and one case of increased SIJ pain (Calodney et al., 2024). Overall, these findings suggest that posterior, minimally invasive, percutaneous techniques are associated with a low incidence of major adverse events.

A summary of the safety, revision rates, and fusion can be found in Table 3.

**Table 3:** Summary of safety, revision, and fusion of SIJF approaches.

Approach	Reported Complications	Revision Rates	Fusion Assessment
Lateral	Most extensively reported on including: Post-procedural pain, hematoma, wound complications, infection, transient neuropathic symptoms, cortical breach, vascular and hardware-related complications	Low to moderate based on pool meta-analysis and cohort data, varying across studies and follow-up duration	Higher reported fusion rates; definitions of fusion and imaging criteria vary across studies.
Posterior	Low reported complications such as graft migration/loosening, but the literature is sparse.	Low but limited by small sample sizes, short-term follow-ups, and the total number of studies.	Moderate to high reported fusion rates with limited studies; definitions of fusion and imaging criteria vary across studies.
Posterolateral	Low reported complications such as screw migration/loosening, but the literature is sparse.	Low but limited by small sample sizes, short-term follow-ups, and the total number of studies.	High reported fusion rates with limited studies; definitions of fusion and imaging criteria vary across studies.

## 5 Discussion

### 5.1 Device manufacturers and markets

It's important to note that the majority of the SIJF literature is based in North America, with less literature internationally. The market for minimally invasive SIJF has expanded rapidly over the past decade, coinciding with the recognition of the SIJ as a clinically significant source of chronic LBP. The growth has been accompanied by multiple platforms and surgical approaches, involving lateral, posterior, and posterolateral techniques. As procedural utilization has increased, the SIJF landscape has become diverse, with the US FDA approving more than 20 device systems, the majority of which correspond with the lateral transiliac approach (Kaye et al., 2024). This rapid diversification has outpaced that of long-term, comparative data, contributing to variability in how devices are adapted and evaluated.

According to the FDA implications, these devices are deemed appropriate for patients suffering from chronic SIJ pain or traumatic and degenerative disruption of the joint (Kaye et al., 2024). Notably, many devices that are commonly employed for SIJF are categorized as unclassified allograft bone products derived from human cells and tissues. Due to this regulatory classification, the FDA does not provide a specific indication statement for SIJF (Kaye et al., 2024). This unique framework enables multiple device platforms to enter the market through varying regulatory pathways, without uniform SIJF indication requirements, which can complicate device-level comparison and the standardization of evidence across manufacturers. While the 510(k) pathway can lead to increased innovation and patient access, it has also led to the rapid expansion of available SIJF devices despite limited comparative and independently funded studies. This rapid proliferation across a short time period raises questions about the relationship between innovation, market trends, and clinical evidence. The advent of minimally invasive SIJF has led a signficant decline in the number of open SIJF as it appears that the surgical community prefers minimally invasive routes in the best interest of their patients. The International Society for the Advancement of Spine Surgery and Society for Minimally Invasive Spine Surgery conducted a survey of their members who performed at least one open or minimally invasive SIJF between 2009 and 2012 and found that the percentage of minimally invasive SIJF increased from 39% in 2009 to over 87% in 2012 (Lorio et al., 2014). This trend has continued to increase as between 2010 and 2021, the annual volume of SIJF grew by 33.5% on average, with a projected annual percent change of 53.3% over the next four years (Waters et al., 2025). Similarly, from 2007 to 2021, the volume of minimally invasive SIJF increased by 258% by spine surgeons while nonsurgeon volume increased by 990.9% (Johnstone et al., 2025). Although this increase in procedural adoption may reflect a growing recognition of the SIJ as the pain generator for pathology, it is still unclear if high-quality independent studies will match the pace of these utilization trends.

Within this regulatory environment, manufacturers have focused on innovation in implant geometry. One distinguished development has been the introduction of 3D-printed porous TTIs, designed to promote osseointegration through controlled lattice structures and increased surface area. Post-market surveillance evaluating these implants included more than 5000 commercially implanted devices, with a reported cumulative probability of surgical revision of less than 2% at one year and no documented cases of implant breakage or migration (Cher et al., 2018). When compared with earlier machined implant designs, 3D-printed implants showed similar rates of complaints and adverse events, suggesting that additive manufacturing has not introduced new short-term safety concerns (Cher et al., 2018). However, this data is derived from manufacturer-maintained surveillance systems and relies on voluntary reports, which may limit the completeness and independence of safety assessments.

Despite the favorable safety profiles, the broader evidence base supporting SIJF devices remains constrained by heterogeneity and industry sponsorships. A systematic review of minimally invasive SIJF identified fewer than 20 primary clinical studies, the majority of which were prospective or retrospective cohort studies rather than RCTs (Lee et al., 2021). The authors noted substantial variability in study design, outcome measures, follow-up duration, and reporting standards, with most studies supported or sponsored by industry manufacturers (Lee et al., 2021). While industry collaboration plays an important part in developing and evaluating SIJF approaches and technologies, sponsorship inherently introduces the potential for publication bias and selective outcome reporting. The majority of the current literature consists of single-arm studies or post-market surveillance, which limits the ability to assess comparative effectiveness across approaches and devices. Thus, positive results should be interpreted within the context of the methodological limitations and possible risks of bias.

In combination with the reliance on post-market surveillance data, these patterns call attention to industry-related evidence gaps, particularly in independent comparative effectiveness research and long-term biomechanical validation. As the SIJF market continues to expand, higher-quality independent studies with standardized reports and lengthened follow-ups will be essential to clarify the comparable contributions of different device designs, manufacturing strategies, and regulatory classifications for clinical outcomes.

### 5.2 Future directions

The major limitation currently in the literature is the lack of validated criteria for SIJF. As a result, definitions vary significantly across studies. This lack of consensus can also be seen broadly across radiological assessments in spinal fusion. An important issue within the literature is the distinction between true arthrodesis and mechanical stabilization for SIJF. Many current devices and marketing promote minimally invasive SIJF as fusion technologies, but the current evidence towards osseointegration is limited, and radiographic definitions for successful fusions vary. Even for studies that use established scoring systems, there is still inconsistent use of fusion criteria (Bilancia et al., 2025). Thus, future studies in SIJF should focus on standardizing objective radiological criteria to improve reliability and clinical assessment of arthrodesis. Additionally, a consensus criterion should be established to differentiate between implant-mediated mechanical stabilization and true arthrodesis. These standardized criteria may incorporate both clinical and radiographical endpoints. Clinical outcome criteria should utilize validated patient-reported outcomes (e.g., VAS, NRS, ODI), return to activity, duration of symptom improvements, and survival rates (no revision surgeries). Radiographical criteria may include CT confirmation of bone bridging across the SIJ, absence of implant loosening/migration, and reproducible fusion definitions validated by independent reviewers. This standardization can allow for meaningful comparisons between devices and approaches and the interpretation of fusion rates. Finally, we suggest that terminology should be adopted to reflect the underlying mechanism of minimally invasive SIJF. For example, for cases where SIJF does not demonstrate osseous bridging but does achieve motion reduction with symptom relief, this can be termed “SIJ stabilization” or “implant fixation” rather than arthrodesis. Consensus terminology would help decrease ambiguity and improve transparency.

However, another important issue within the literature is the relationship between reported fusion rates and clinical success. Current literature has reported that meaningful improvements in pain and function are demonstrated despite the limited evidence in osseointegration across the SIJ, which suggests that mechanical stabilization may be a significant factor for symptom relief. Thus, if the primary mechanism is stabilization rather than true arthrodesis, the long-term outcomes are uncertain as implant loosening/migration or other sources of mechanical failure points could theoretically decrease this stabilizing effect over time. However, this relationship is not currently elucidated with long-term data. Additionally, reports of implant safety rely on manufacturer registeries or post-market surveillance databases which provide real-world information, but have possible risks of bias such as underreporting, reporting bias, and selection bias. As a result, the true incidence of long-term implant loosening/migration, and revision rates may be hard to determine. Thus, future studies should utilize independent surveillance methods as well to help determine if outcomes are maintained at varying follow-up lengths in the absence of or incomplete fusion.

Another focus for future studies should be conducting independently funded RCTs that compare SIJF to conservative treatments, interventional options such as radiofrequency, and placebo/sham procedures. Current evidence on the SIJFs, particularly the posterior and posterolateral approaches, is limited by short-term follow-up; there is limited extended follow-up beyond 2 years (Gill et al., 2025). There is also a need for direct head-to-head comparisons between these three approaches with long-term follow-ups to determine efficacy, fusion rates, and long-term durability. Furthermore, the rapid entry of many new SIJF devices into the market underscores and calls attention to the influence of market pressures within the field. We suggest strengthening the 510k pathway (especially the “substantial equivalence” evidence for approval) and requiring more stringent clinical evidence to ensure that new devices that are adopted follow evidence-based medicine rather than the market. Additionally, future research should examine the impact of regulatory pathways and geographic SIJF practice patterns to assess the long-term evidence of SIJF and whether these patterns are reflected in other geographic regions.

Additionally, most studies looked at normal bone or osteopenic bone, not osteoporotic bone. Further research should be conducted in cohorts or models with osteoporosis to assess efficacy in higher-risk populations (Raji et al., 2024).

## 6 Conclusion

Minimally invasive SIJF has become an increasingly utilized intervention for select patients with chronic SIJ-mediated pain who fail conservative management. Advances in surgical techniques and implant technologies have expanded the available treatment options, particularly with the emergence of posterior and posterolateral approaches that aim to reduce soft tissue disruption and neurovascular risk. Current evidence supports meaningful improvements in pain, function, and quality of life across multiple minimally invasive SIJF techniques, with generally favorable safety profiles.

Despite these encouraging outcomes, the existing literature is characterized by notable limitations. Many studies are industry-sponsored, employ heterogeneous methodologies, or lack long-term follow-up, making it difficult to draw definitive conclusions regarding comparative effectiveness and durability. Biomechanical data further suggest that most minimally invasive SIJF procedures achieve partial stabilization rather than true bony fusion, raising important questions about terminology, expected mechanisms of benefit, and long-term joint behavior. Additionally, patient-specific factors such as anatomical variability, bone quality, and prior spinal surgery remain underexplored in current outcome studies.

To advance the field, future investigations should focus on rigorous, head-to-head comparisons of lateral, posterior, and posterolateral approaches using standardized outcome measures and extended follow-up periods. Greater emphasis on biomechanical validation, patient selection criteria, and performance in higher-risk populations, including those with osteoporosis, is needed. Addressing these evidence gaps will be essential for refining surgical indications, optimizing technique selection, and ensuring that the continued growth of minimally invasive SIJF is guided by robust, high-quality evidence rather than technological momentum alone.

## Ethics approval and consent to participate

Not applicable.

## Consent for publication

Not applicable.

## Availability of data and materials

No/Not applicable. This manuscript does not report data generation or analysis.

## Authors’ contributions

JW, AA, NP, AK, JS, and FE contributed to the conception and design of the study. All authors acquired, analyzed, and interpreted the data. All authors contributed to drafting, revising, approving, and accountable for the submitted manuscript.

## Funding

All authors declare no funding was received.

## Conflict of interest

All authors declare no potential competing financial interests or personal relationships as specified on required ICMJE Disclosure Forms.

## Glossary

SI: sacroiliac; SIJ: sacroiliac joint; LBP: low back pain; NSAIDS: non-steroidal anti-inflammatory drugs; SIJF: sacroiliac joint fusion; FDA: food and drug administration; CT: computed tomographic; TTI: triangular titanium implant; DBM: demineralized bone matrix; PTI: porous threaded titanium implants; TDI: triangular dowel implant; CDI: cylindrical threaded implants; PSIS: posterior superior iliac spine; PAK: pedicle access kit; PLTI: posterolateral transiliac; VAS: visual analog scale; ODI: Oswestry Disability Index; NRS: numerical rating scale; MME: morphine milligram equivalents; RCT: randomized controlled trials.
